# Personal

**Published:** 1890-07

**Authors:** 


### Personal.

Dr. S. S. Green has removed to No. 326 Niagara street. Office hours: 2 to 3 and 7 to 8 P. M.

---

Dr. Eli H. Long has been appointed Professor of Materia Medica in the department of pharmacy of the University of Buffalo, N. Y.

---

Dr. John R. Gray has been appointed to fill the professorship of Pharmacognosy in the same institution.

---

Dr. William H. Parish was recently appointed Professor of Gynecology in the newly created chair in the Philadelphia Polyclinic Hospital.

---

Dr. Charles A. Wall has removed to 306 Hudson street, corner Plymouth avenue, Buffalo, N. Y. Office hours: 9 A. M., 2 and 7 P. M. Telephone No. 1637.

---

Dr. Wm. C. Krauss, of Buffalo, was elected a member of the American Neurological Association at the annual meeting recently held in Philadelphia. On his way there Dr. Krauss delivered a lecture at Cornell University on The Histological Investigation of the Nervous System, and was made non-resident lecturer in that department.

---

Dr. Neil L. McPhatter, formerly of Guelph, who settled in Cleveland after his return from Birmingham, where he worked six months with Mr. Lawson Tait, has gone to Denver, Colorado, where he is engaged in a large and lucrative practice. He has the chair of Gynecology in the university of that city.—*Canadian Practitioner.*

---

Dr. George T. Wetmore, of New York City, is visiting the physicians through northern and central New York as the representative of the Physicians' Mutual Aid Association. We have heretofore earnestly recommended this society to our readers, and now again urge them to secure its benefits when the opportunity is thus favorably offered.

---

Dr. Joseph M. Mathews, of Louisville, Ky., one of the most accomplished surgeons south of the Ohio river, came within four votes of election to the presidency of the American Medical Association at Nashville. Dr. Mathews is a presiding officer of rare accomplishments, is one of the most eloquent and effective speakers within the ranks of American medical profession, and is fitted by education, culture and by all the attainments that go to make up a well-poised character, to wield the gavel of the Association, and we hope to see him do so at no distant day. The *Mediial Mirror* for June publishes a fine photogravure of this distinguished physician.

